# The multimorbidity collaborative medication review and decision making (MyComrade) study: a pilot cluster randomised trial in two healthcare systems

**DOI:** 10.1186/s40814-022-01107-y

**Published:** 2022-10-04

**Authors:** Collette Kirwan, Lisa Hynes, Nigel Hart, Sarah Mulligan, Claire Leathem, Laura McQuillan, Marina Maxwell, Emma Carr, Kevin Roche, Scott Walkin, Caroline McCarthy, Colin Bradley, Molly Byrne, Susan M. Smith, Carmel Hughes, Maura Corry, Patricia M. Kearney, Geraldine McCarthy, Margaret Cupples, Paddy Gillespie, Anna Hobbins, John Newell, Liam Glynn, Davood Roshan, Carol Sinnott, Andrew W. Murphy

**Affiliations:** 1 Health Research Board Primary Care Clinical Trials Network Ireland, National University of Ireland, Galway, Ireland; 2 College of Medicine, Nursing & Health Sciences, National University of Ireland, Galway, Ireland; 3 Croí, West of Ireland Cardiac and Stroke Foundation, Galway, Ireland; 4 School of Medicine, Dentistry & Biomedical Sciences, Queen’s University, Belfast, Northern Ireland; 5 Sligo Medical Academy, National University of Ireland, Galway, Ireland; 6 Northern Ireland Clinical Research Network (Primary Care), Belfast, Northern Ireland; 7 School of Allied Health, University of Limerick, Limerick, Ireland; 8 Department of General Practice, RCSI University of Medicine and Health Sciences, Dublin, Ireland; 9 Department of General Practice, University College Cork, Cork, Ireland; 10 Health Behaviour Change Research Group, School of Psychology, National University of Ireland, Galway, Ireland; 11 General Practice, Discipline of Public Health & Primary Care, Trinity College, Dublin, Ireland; 12 School of Pharmacy, Queen’s University, Belfast, Northern Ireland; 13 General Practice Federation, Belfast, Northern Ireland; 14 School of Public Health, University College Cork, Cork, Ireland; 15 Mental Health Services, Health Services Executive, Sligo, Ireland; 16 Health Economics & Policy Analysis Centre, National University of Ireland, Galway, Ireland; 17 CURAM, Science Foundation of Ireland (SFI) Research Centre for Medical Devices, National University of Ireland, Galway, Ireland; 18 School of Mathematical & Statistical Sciences, National University of Ireland, Galway, Ireland; 19 School of Medicine, University of Limerick, Limerick, Ireland; 20 THIS Institute, University of Cambridge, Cambridge, UK

## Abstract

**Background:**

While international guidelines recommend medication reviews as part of the management of multimorbidity, evidence on how to implement reviews in practice in primary care is lacking. The MyComrade (MultimorbiditY Collaborative Medication Review And Decision Making) intervention is an evidence-based, theoretically informed novel intervention which aims to support the conduct of medication reviews for patients with multimorbidity in primary care.

**Aim:**

The pilot study aimed to assess the feasibility of a definitive trial of the MyComrade intervention across two healthcare systems (Republic of Ireland (ROI) and Northern Ireland (NI)).

**Design:**

A pilot cluster-randomised controlled trial was conducted (clustered at general practice level), using specific progression criteria and a process evaluation framework.

**Setting:**

General practices in the ROI and NI.

**Participants:**

Eligible practices were those in defined geographical areas who had GP’s and Practice Based Pharmacists (PBP’s) (in NI) willing to conduct medication reviews. Eligible patients were those aged 18 years and over, with multi morbidity and on ten or more medications.

**Intervention:**

The MyComrade intervention is an evidence-based, theoretically informed novel intervention which aims to support the conduct of medication reviews for patients with multimorbidity in primary care, using a planned collaborative approach guided by an agreed checklist, within a specified timeframe.

**Outcome measures:**

Feasibility outcomes, using pre-determined progression criteria, assessed practice and patient recruitment and retention and intervention acceptability and fidelity. Anonymised patient-related quantitative data, from practice medical records and patient questionnaires were collected at baseline, 4 and 8 months, to inform potential outcome measures for a definitive trial. These included (i) practice outcomes—completion of medication reviews; (ii) patient outcomes—treatment burden and quality of life; (iii) prescribing outcomes—number and changes of prescribed medications and incidents of potentially inappropriate prescribing; and (iv) economic cost analysis. The framework Decision-making after Pilot and feasibility Trials (ADePT) in conjunction with a priori progression criteria and process evaluation was used to guide the collection and analysis of quantitative and qualitative data.

**Results:**

The recruitment of practices (*n* = 15) and patients (*n* = 121, mean age 73 years and 51% female), representing 94% and 38% of a priori targets respectively, was more complex and took longer than anticipated; impacted by the global COVID-19 pandemic. Retention rates of 100% of practices and 85% of patients were achieved. Both practice staff and patients found the intervention acceptable and reported strong fidelity to the My Comrade intervention components. Some practice staff highlighted concerns such as poor communication of the reviews to patients, dissatisfaction regarding incentivisation and in ROI the sustainability of two GPs collaboratively conducting the medication reviews. Assessing outcomes from the collected data was found feasible and appropriate for a definitive trial. Two progression criteria met the ‘Go’ criterion (practice and patient retention), two met the ‘Amend’ criterion (practice recruitment and intervention implementation) and one indicated a ‘Stop – unless changes possible’ (patient recruitment).

**Conclusion:**

The MyComrade intervention was found to be feasible to conduct within two different healthcare systems. Recruitment of participants requires significant time and effort given the nature of this population and the pairing of GP and pharmacist may be more sustainable to implement in routine practice.

**Trial registration:**

Registry: ISRCTN, ISRCTN80017020; date of confirmation 4/11/2019; retrospectively registered.

**Supplementary Information:**

The online version contains supplementary material available at 10.1186/s40814-022-01107-y.

## Key messages regarding feasibility

- What uncertainties existed regarding the feasibility?
  The acceptability of the MyComrade intervention to practices and patients was uncertain. The feasibility of including medication reviews in daily clinical practice was unknown as were the relative merits of medication reviews by dyads of GP:GP or GP:PBP.
- What are the key feasibility findings?
  The study has demonstrated the feasibility of practice and patient retention, intervention implementation and outcome assessments. An ADePt evaluation allowed the identification of appropriate modifications for future trial design, particularly around practice and patient recruitment.
- What are the implications of the feasibility findings for the design of the main study?
  A definitive trial is possible with modifications to recruitment, ensuring structured feedback to patients and greater consideration towards incentivising medication reviews in primary care. Careful consideration is required regarding the relative merits of GP:GP or GP:PBP dyads.

## Introduction

The increasing prevalence of multimorbidity (the co-occurrence of two or more long-term conditions) and polypharmacy (five or more medications) is a cause of concern to patients, practitioners and policymakers [28]. Best practice guidelines regarding the clinical management of patients with multimorbidity and polypharmacy recommend at least an annual medication review and more often if indicated, for example subsequent to hospital admissions [30, 31]. The National Institute for Health and Care Excellence in the UK (NICE) highlights the use of screening tools to identify medicine-related safety concerns and to consider de-prescribing when benefits no longer outweigh harms [31]. However, reviews of interventions aiming to improve outcomes for patients with multimorbidity in primary care noted a limited evidence base with interventions having mixed effects [46].

The MultimorbiditY Collaborative Medication Review And Decision Making (MyComrade) Intervention is a novel approach to medication reviews for patients with multimorbidity which was designed and tested in line with the Medical Research Council (MRC) Framework for the development and evaluation of complex interventions [40–42]. In-depth descriptions of the MyComrade intervention development and early feasibility testing are available elsewhere [23, 40]. This study builds on the findings of the MyComrade feasibility study in which ten general practices operating in the southwest of Ireland participated [40].

## Aims and outcomes

The aim of this study was to evaluate the feasibility of a trial of the MyComrade intervention across two healthcare systems using a pilot cluster randomised controlled trial (cRCT). The objectives were to:

1. Determine the feasibility of a definitive trial of the MyComrade intervention, focusing on (a) recruitment, (b) retention and (c) fidelity of intervention implementation. These were assessed using predetermined progression criteria [3, 23].
2. Select (a) suitable outcome(s) and (b) cost-effectiveness measures for use in a definitive trial. Key trial outcome measures included an evaluation of the conduct of medication reviews, patient self-reported quality of life and treatment burden questionnaires, prescription changes and potential inappropriate prescribing, in addition to cost analyses. These are further detailed below and in Appendix 1.

We utilised the process of Decision-making after Pilot and feasibility Trials (ADePT) to structure the reporting on methodological issues and to allow identification of appropriate modifications for a future trial design [5].

## Methods

The study protocol has been detailed elsewhere [23] (ISRCTN80017020)—a summary of which is provided here. Adaptations to the protocol applied during the pilot study are also described.

### Trial design

A pilot cRCT of the MyComrade intervention was undertaken between January 2020 and March 2021. General practices were the units of randomisation (the clusters) and individual patients with multimorbidity, prescribed 10 or more medications, were the units of analysis (the patients). A cluster design was chosen as the intervention was applied by the general practice.

### Study population and setting

This study took place in general practices in Northern Ireland (NI) and the Republic of Ireland (ROI). The populations of these jurisdictions are similar in terms of ethnicity [33] and socioeconomic gradients [32]. GPs in both jurisdictions work as independent contractors [49] but the health systems differ in important ways, principally that the system in ROI is a mixed public and private system, while the system in NI is publicly funded [10].

Since 2016, most GP practices in NI have access to a Practice Based Pharmacist (PBP), although the hours and role of the PBP vary depending on the size and specific needs of the practice. Tasks performed by the PBP may include medication reviews and medication reconciliation following discharge from hospital [50]. There is no similar access to a PBP in ROI. This study was therefore designed to assess medication reviews conducted by two GPs in the ROI and by a GP and PBP in NI.

### Eligibility criteria and recruitment

Practice and patient recruitment commenced in July 2019 and was completed in May 2020. The protocol specified that 320 patients would be recruited from 16 practices [23].

#### Practices

As specified by INTERREG (www.interregeurope.eu/), a European inter-regional cooperation agency and the funder for this trial, all practices in NI were eligible. In ROI general practices were eligible if based in any of the contiguous counties along the border between ROI and NI. The general practices required 2 GPs in ROI and 1 GP and 1 PBP in NI willing to conduct medication reviews. Practices were excluded if involved in other multimorbidity-related research trials.

NI has a national registry of general practices with email address contacts; practices were contacted by email only. ROI does not have a similar list. The recruitment process in ROI therefore included a variety of recruitment strategies: email using either personal contacts or addresses obtained from the HRB Primary Care Clinical Trials Network of Ireland (https://primarycaretrials.ie/), postal mail, cold calling and presenting information about the study at GP training events and meetings.

#### Patients

Practice staff conducted patient screening to assess eligibility, which involved two steps: an initial rapid electronic screening for patients, as per study protocol inclusion/exclusion criteria [23], who were prescribed ten or more medications and were over 18 years of age, followed by a more specific screening of this list for patient exclusion criteria relating to a terminal illness, pregnancy, cognitive or learning disabilities that would prevent them from completing the study activities.

Recruitment packs were mailed to patients by practice staff. This pack included a cover letter, patient information leaflet, consent form and baseline questionnaires. Potential patients were encouraged to contact their GP or research staff if they had any queries and had 1 month from the time of posting of the pack to return the consent form and baseline questionnaire. Included patients completed a written consent form, which was posted to research staff, using a provided stamped addressed envelopes.

### Intervention

The intervention, informed by the COM-B (Capability, Opportunity, Motivation – Behaviour) model of behaviour change and Behaviour Change Technique (BCT) Taxonomy [27], aims to support general practitioners (GPs) to conduct medication reviews for patients with multimorbidity with a view to optimising the medication regimen and minimising potentially inappropriate prescribing. The study utilised the revised operationalisation of BCTs in the MyComrade intervention, based on findings of an earlier feasibility study [40] and are detailed in the MyComrade study process evaluation and outcomes framework [23], see Table 1.

**Table 1:** MyComrade intervention: barriers to medication reviewing and operationalisation of MyComrade BCTs

***Behavioural analysis using COM-B***	***MyComrade intervention*** ***Behaviour change technique and definition***	***Revised operationalisation of BCTs in the MyComrade intervention, based on findings of feasibility study(Sinnott et al., *** ***2017) and contextual requirements***
***Psychological capability***	3.2 Social support – practical Advise on, or provide practical help (e.g. colleagues) for performance of behaviour	Two GPs, or a GP and PBP collaboratively conduct medication review
***Social opportunity***		
***Physical opportunity***	12.2 Restructuring social environment Change, or advise to change the social environment in order to facilitate performance of the wanted behaviour 1.4 Action planning Prompt detailed planning of performance of behaviour (must include at least one of frequency, context, duration, intensity)	Plan active medication reviews
***Automatic motivation***	7.1 Prompts/cues Introduce environmental or social stimulus for the purpose of prompting or cueing behaviour	Use of prescribing checklist
***Reflective motivation***	10.7 Self-incentive Plan to reward self in future if and only if there has been effort and/or progress in performing the behaviours	Annual professional appraisal contribution
***Reflective motivation***	10.1 Material incentive (Behaviour) *Inform that money, vouchers or other valued objects will be delivered if and only if there has been effort and/or progress in performing the behaviour*	Financial reimbursement *All practices:* €500/£430 for patient recruitment admin costs *Intervention practices only:* ● €100/£86 completion intervention training ● €50 /£43 per completed medication review

The revised MyComrade intervention differed from the earlier feasibility study (Sinnott, 2017) due to the significant inclusion of practice-based pharmacists in NI as collaborative reviewers. In addition, incentives were incorporated into the intervention to compensate practices for the high opportunity costs spent in conducting both the study and medication reviews. This consisted of €500 (£430) to practices for agreeing to participate in the study, €100 (£86) for each health care professional (HCP) who participated in the intervention training and €50 (£43) per completed medication review. The revised intervention is further described using the Template for Intervention Description and Replication (TIDieR) Checklist [21] (see Appendix 2).

Intervention practices received MyComrade intervention training either face-to-face from research team members (SM, LH, LMcG) or using pre-recorded material, depending on practice preference. The training sessions were audio-recorded to allow assessment of fidelity in terms of content and duration.

Training materials emphasised that while the implementation of the intervention could be adapted within practices, it was important to conduct the review collaboratively using NO TEARS checklist [25], to engage in shared decision making with the patients after reviews, and to upload the review notes into the GP practice software system for data collection purposes.

### Process evaluation

A process evaluation [23] informed by an approach described by Grant et al. (2012) and Proctor et al.’s [34] taxonomy of implementation outcomes (acceptability, adoption, appropriateness, feasibility, fidelity, implementation cost, coverage and sustainability) was conducted. The main purpose of the process evaluation is to answer questions relating to the primary feasibility outcomes (recruitment, retention and intervention implementation) identified in the predetermined progression criteria, using qualitative and quantitative data [23].

### Progression criteria

The progression criteria comprised pre-defined ‘Stop/Amend/ Go’ criteria [3] and were developed by team consensus a priori [23]. These criteria were:

1. Practice recruitment—Can 16 practices be recruited in 3 months (8 NI/8 ROI)?
2. Patient recruitment—Can 20 patients per practice be recruited (total = 320)?
3. Practice retention—Can ≥ 14 practices be retained until study end?
4. Patient retention—Can at least 80% of recruited patients be retained until study end?
5. Intervention implementation—Was delivery of the intervention judged feasible by findings from qualitative data?

The decision to progress to a full trial drew on an evaluation of quantitative and qualitative data using the ADePT process [5], discussions within the research team and the Patient and Public Involvement (PPI) Panel. The ADePT process includes 14 methodological issues when reporting trial feasibility including sample size estimation, recruitment, consent, intervention adherence, intervention acceptability, costs and duration, completion and appropriateness of outcome assessments, retention, logistics and synergy between protocol components, with an emphasis on understanding the issue and developing solutions for the issue.

### Outcomes

The key trial outcomes, based on a 2018 Core Outcome Set for multimorbidity research [47], assessing the impact and responsiveness to the MyComrade intervention, included:

- Practice outcomes: Number of medication reviews conducted
- Patient outcomes: Patient completed multimorbidity treatment burden (MTBQ) [13] and quality of life (EQ-5D-3L) [16] questionnaires at baseline and 4 and 8 months.
- Prescribing outcomes: Indicators of potentially inappropriate prescribing (PIPs) [2] (see Appendix 3), the number of prescribed medications and rates of deprescribing, collected from medical records at baseline and 4 and 8 months.
- Economic cost outcomes: health service utilisation collected from medical records at baseline and 4 and 8 months, quality of adjusted life years (EQ-5D-3L) and intervention costs.

A full list of the collected data and outcome measures are presented in Appendix 1.

### Data collection and analysis

#### Quantitative

SPSS (version 17) was used for data entry and management, while the R programming language (version 4.1.0) was used for all analyses to compare outcomes between groups, and an accompanying sample size calculation for the definitive trial. Suitable numerical summaries (e.g. mean and standard deviation for continuous variables and frequency tables for categorical variables) were generated to summarise the key variables of interest. All analyses adhered to statistical best practice following the principles of reproducible research.

#### Qualitative

Semi-structured interviews were conducted with a purposive sample of participating practice staff (GP, PBP and administrative staff) and patients. Interviews focused on interviewees’ experiences of multimorbidity management, the feasibility and acceptability of the My Comrade intervention and were conducted via telephone, audio-recorded, transcribed verbatim and analysed in QSR NVivo 12 [35]. Interview schedules, while following same general format, were customised for practice staff and patients, guided by input from pilot interviews outcomes and the MyComrade PPI panel. The interview data were analysed using a framework approach [18, 37] which was guided by the My Comrade process evaluation framework (described above). The PPI panel inputted into the qualitative analysis and write up.

#### Health economic analysis

An analysis of the feasibility of conducting a health economic evaluation as part of a future definitive trial was undertaken. The healthcare perspective was adopted with respect to costs, presented in 2020 Euro prices, and health outcomes were expressed as quality-adjusted life years (QALYs), based on the EQ-5D-5L instrument. The cost of the MyComrade intervention in ROI and NI was estimated. Costs relating to healthcare usage were estimated, based on data from the trial and unit cost estimates for ROI and NI (C.S.O., 2020; H.I.Q.A., 2020; P.S.S.R.U., 2020).

### Public and patient involvement

A cross-border PPI panel, established early 2020, involved four adults (two women and two men), three from NI and one from ROI, who were living with and/or caring for someone with multimorbidity. The establishment and facilitation of this group as research partners was guided by the PPI Ignite @ NUI Galway programme office, part of a national PPI initiative. Due to COVID, the once to twice monthly interactions with the PPI panel were convened over Zoom and via email. The group contributed to the development of study documents and interview schedules, data analysis and dissemination.

### Randomisation

A biostatistician blinded to allocation randomised practices in the ratio 1:1 using an online system ‘Sealed Envelope’. To limit recruitment bias, patient recruitment ceased prior to randomisation of relevant practices.

## Results

Fifteen practices and 121 patients participated representing 94% and 38% of a priori targets respectively. Their profiles are detailed in Table 2 General practice profile and Table 3 Patient profile.

**Table 2:** General practice profile

**General practice profile**		**Control (*n* = 7)**	**Intervention (*n* = 8)**	**Total (*n* = 15)**
***Location***	Rural	3 (43%)	2 (25%)	5 (33%)
	Urban	2 (29%)	3 (38%)	5 (33%)
	Mixed	2 (29%)	3 (38%)	5 (33%)
*H****ealth Information System***	EMIS (NI only)	2 (29%)	0 (0%)	2 (13%)
	Vision (NI only)	1 (14%)	4 (50%)	5 (33%)
	Helix (ROI only)	2 (29%)	1 (12%)	3 (20%)
	Socrates (ROI only)	2 (29%)	0 (0%)	2 (13%)
	HealthOne (ROI only)	0 (0%)	3 (38%)	3 (20%)
***Number of full-time***^***a***^*** GPs**** mean (SD)*		2.9 (0.90)	2.1 (1.1)	2.5 (1.1)
***Number of part-time GPs**** mean (SD)*		2.1 (2.4)	1.3 (± 1.0)	1.7 (1.8)
***Number of GP Session/practice**** mean (SD)*		44 (21)	23 (8.7)	33 (19)
***Number of practice nurse session/practice**** mean (SD)*		14 (8.6)	9.0 (3.4)	11 (6.7)
***GP practice size (number of patients registered)***	< 2,500	0 (0%)	1 (12%)	1 (7%)
	> 2,500 < 5,000	2 (29%)	5 (62%)	7 (47%)
	> 5,000 < 7,500	0 (0%)	1 (12%)	1 (7%)
	> 7,500	5 (71%)	1 (12%)	6 (40%)
***Repeat prescribing policy***	Yes	5 (71%)	6 (75%)	11 (73%)
	No	2 (29%)	2 (25%)	4 (27%)
***Time spent on repeat prescribing as per practice policy**** mean (SD)*^*b*^		34 (± 16)	45 (± 35)	40 (± 28)
***Routine medication review***	Usually not	4 (57%)	6 (75%)	10 (67%)
	Almost always	3 (43%)	2 (25%)	5 (33%)
***Annual face-face medication review***	Almost always	5 (71%)	7 (88%)	12 (80%)
	Usually not	2 (29%)	1 (12%)	3 (20%)

^a^ Pragmatically decided as per GP practice

^b^ As per checklists

**Table 3:** Patient profile

**Patient profile**		***Control (n* = *62)***	***Intervention (n* = *59)***	***Total (n* = *121)***
***Age****, mean (sd)*	Years	73 (± 12)	73 (± 10)	73 (± 11)
***Sex****, mean (sd)*	Female	24 (39%)	38 (64%)	62 (51%)
	Male	38 (61%)	21 (36%)	59 (49%)
***Distance from GP****, mean (sd)*	Kilometres	4.4 (± 3.7)	5.4 (± 5.0)	4.9 (± 4.3)
***Level of education****, mean (sd)*	Primary education	13 (21%)	9 (15%)	22 (18%)
	Some secondary education	19 (31%)	19 (32%)	38 (31%)
	Complete secondary	8 (13%)	12 (20%)	20 (17%)
	Some third level	6 (10%)	9 (15%)	15 (12%)
	Complete third level	15 (24%)	10 (17%)	25 (21%)
	No schooling	1 (2%)	0 (0%)	1 (1%)

**Fig. 1:**
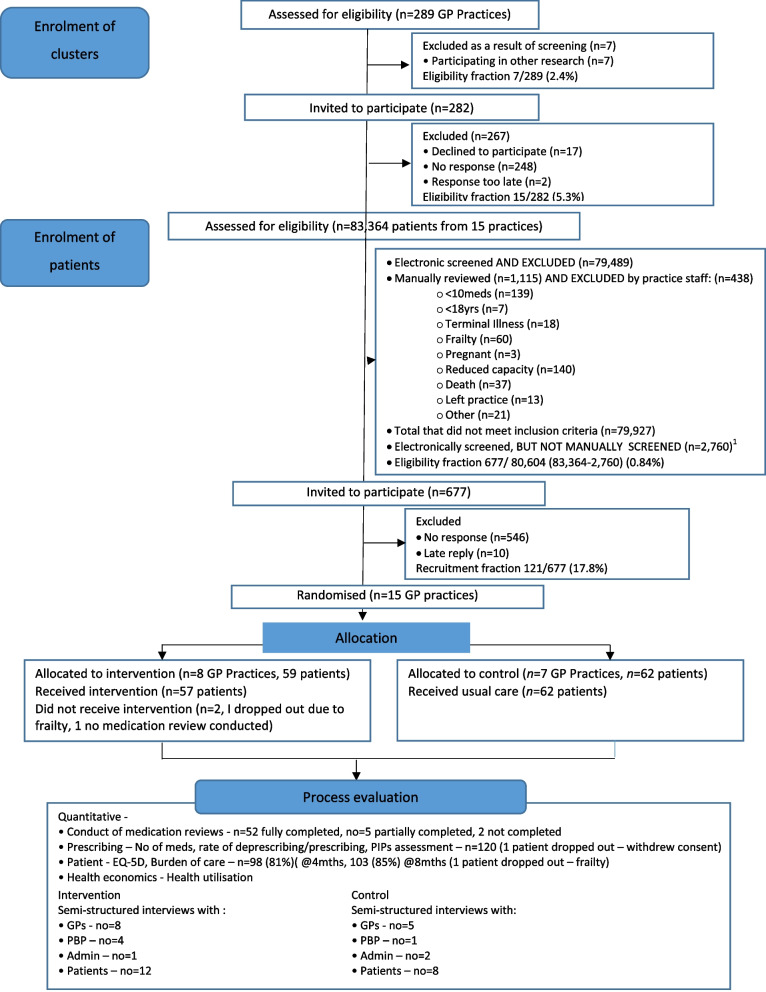
MyComrade Pilot Trial CONSORT flow diagram

The CONSORT flow diagram for participant screening, recruitment and attrition is illustrated in Fig. 1. We report our findings in accordance with the CONSORT extension to pilot and feasibility trials [15] (Appendix 4).

The patient and prescribing outcomes are reported in Tables 4 and 5 respectively. Between control and intervention from baseline to 8 months, there appeared to be minimal changes in quality of life and possibly a small change in favour of the intervention group for treatment burden. There also appeared to be a tentative trend, between baseline and 8 months, towards a higher number of medications being discontinued for patients in the intervention group (1.3 (SD 1.6)) as compared to the control group (0.37 (SD 1.1)) and in medication dose being decreased for patients in the intervention group (0.37 (SD 0.60)) and the control group (0.10 (SD 0.35)). These are obviously very tentative conclusions, requiring a definitive trial, and are further discussed below in the ADePT framework.

**Table 4:** Patient outcomes at 8-month follow-up—EQ-5D-5L and MTBQ Questionnaire summary results

	**Overall difference**		
	**Control (*N* = 62)**	**Intervention (*N* = 59)**	**Total (*N* = 121)**
**Changes in EQ5D—QALY scores from Baseline to 8 months**			
**Mean (± SD)**	0.073 (± 0.56)	− 0.0087 (± 0.48)	0.036 (± 0.52)
**Changes in Treatment Burden scores from Baseline to 8 months**			
**Mean (± SD)**	0.15 (± 0.52)	0.045 (± 0.42)	0.10 (± 0.48)

**Table 5:** Prescription outcome—medication changes over study timeframe

NI and ROI			
	**Control (*N* = 62)**	**Intervention (*N* = 59)**	**Total (*N* = 121)**
**Number of prescribed medications baseline**			
** Mean (± SD)**	12 (± 3.2)	12 (± 2.4)	12 (± 2.8)
** [Min, Max]**	12 [4.0, 22]	12 [9.0, 19]	12 [4.0, 22]
**Number of prescribed medications 8 months**			
** Mean (± SD)**	13 (± 3.3)	12 (± 2.2)	12 (± 2.9)
** Median [Min, Max]**	12 [4.023]	12 [8.0, 19]	12 [4.0, 23]
**Number of new medications**			
** Mean (± SD)**	0.85 (± 1.6)	1.0 (± 1.3)	0.93 (± 1.5)
** Median [Min, Max]**	0 [0, 8.0]	1.0 [0, 6.0]	0 [0, 8.0]
**Number of medications discontinued**			
** Mean (± SD)**	0.37 (± 1.1)	1.3 (± 1.6)	0.80 (± 1.4)
** Median [Min, Max]**	0 [0, 7.0]	1.0 [0, 7.0]	0 [0, 7.0]
**Number of medications dose increased**			
** Mean (± SD)**	0.25 (± 0.68)	0.22 (± 0.45)	0.23 (± 0.58)
** Median [Min, Max]**	0 [0, 3.0]	0 [0, 2.0]	0 [0, 3.0]
**Number of medications dose decreased**			
** Mean (± SD)**	0.10 (± 0.35)	0.37 (± 0.60)	0.23 (± 0.50)
** Median [Min, Max]**	0 [0, 2.0]	0 [0, 2.0]	0 [0, 2.0]

We do not report comparative findings between NI and ROI due to the small sample size.

The fourteen methodological issues outlined in the ADePT tool [5], were mapped to the research aim and objectives. The findings of the ADePT evaluation are summarised in Table 6 and further described below, with quantitative and qualitative evidence from the study provided for each issue. Additional files 1, 2, and 3 present possible solutions to issues identified.

**Table 6:** Summary of main findings for methodological issues identified by the ADePT Framework

Methodological issues	Findings	Evidence
1. Did the feasibility/pilot study allow a sample size calculation for the main trial?	Although dependent on a small sample, sample size calculations were calculated	For example, assuming an average difference of 0.8 medications between intervention and control patients after 8 months, an estimated 172 patients from 26 clusters should be recruited
2. What factors influenced eligibility and what proportion of those approached were eligible?	Eligibility for clusters difficult to determine due to large number of non-responses. For patients, the most common reason for ineligibility at electronic searching stage was not being on ten or more medications and/ or being over 18 years. Other reasons included frailty, terminal illness or death	282 of 289 practices were deemed eligible 677 of 80,604 (83,364 − 2760) patients were deemed eligible
3. Was recruitment successful?	Targets for practice recruitment were met. Targets for patient recruitment were not	15 out of a target of 16 practices recruited (94%), but recruitment took longer than anticipated 121 out of a target of 320 patients recruited within 1 month (38%)
4. Did eligible participants consent?	Low conversion to consent particularly for practices	5.3% (15 out of potential 282 practices) consented 19.3% (131 out of potential 677 participants) consented (121 = 17.9% were eligible to participate)
5. Were participants successfully randomised and did randomisation yield equality in groups?	Randomisation was successful with broadly similar practices and patients	See Tables 3 and 4
6. Were blinding procedures adequate?	Yes for randomisation Not possible for data collection	Blinding with randomisation worked well
7. Did participants adhere to the intervention?	Good adherence to implementation and documentation of the MyComrade Intervention	88.3% (*n* = 52) fully completed checklist
8. Was the intervention acceptable to the participants?	Acceptable for practices with some minor recommendations to change such as incentivisation. Patients were also positive but would like structured/formalised feedback on their medication review	Qualitative data showed that practices recognised the clinical importance of the intervention but raised concerns regarding long-term sustainability. Patients were positive but were concerned with the lack of communication on the medication review
9. Was it possible to calculate intervention costs and duration?	Yes	See Appendix 5
10. Were outcome assessments completed?	Good completion rates of outcome assessments	*Practice based*: 88% and 8% of medication review checklists were fully and partially completed respectively *Patient outcomes*: 81 and 85% of patient questionnaires were returned at 4 and 8 months respectively
11. Were outcomes measured those that were the most appropriate outcomes?	Yes	Outcomes were consistent with the internationally agreed COSmm (Smith, 2018). Inclusion of additional outcomes from the COSmm could be considered in a full trial
12. Was retention to the study good?	Yes	Practice retention was 100% Patient retention 81% (*n* = 98) at 4 months, 85% (*n* = 103) at 8 months
13. Were the logistics of running a multicentre trial assessed?	Yes	Contracts with partner institutions and practices were identified as being resource intensive especially for a definitive trial

### Did the study allow a sample size calculation for the main trial?

Data on the patient and prescribing outcomes, detailed earlier, are suitable for use for future sample size calculations (Tables 5 and 6). For example, if deprescribing was identified as the most appropriate primary outcome (measured using medication counts) then the following calculation would apply.

Using a sample size calculator designed for cluster randomised trials [7, 20] and the data on medication changes from this study (Table 5), supplemented with data from the similar SPPiRE study [26], it is considered appropriate to detect an absolute difference in the number of medications between the two arms of the study, at eight months follow-up, of 0.8. Based on the findings in this study of a standard deviation of the difference in the number of medications from baseline to eight months of 1.29 and an intraclass coefficient (ICC) of 0.049, we estimate that 156 patients from 26 clusters (13 clusters from each arm; 6 patients from each cluster) would be required to detect this difference with 90% power. Allowing conservatively for dropouts of 10% (as compared to the 1% reported here), a definitive trial would require a total sample size of 172 (7 patients from each cluster).

**Fig. 2:**
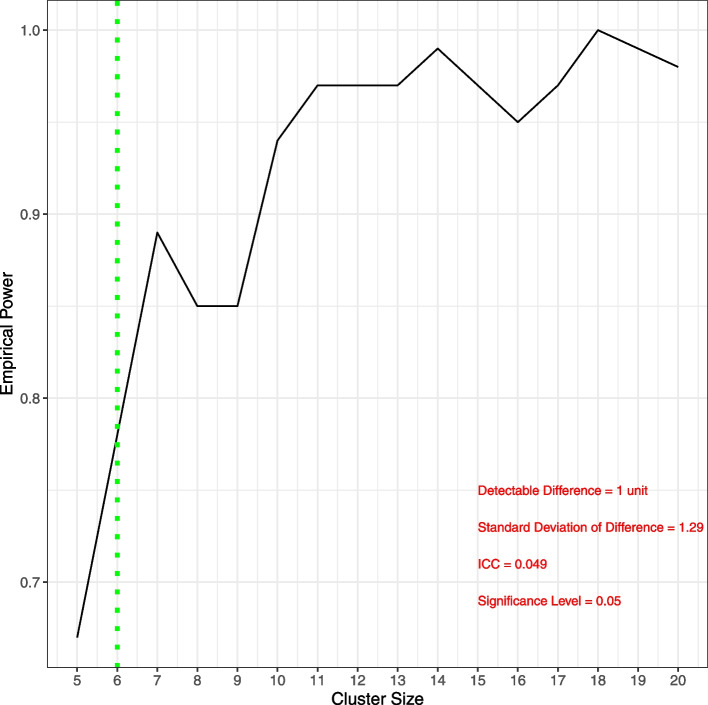
Cluster size power

A simulation study [19] was also performed to assure an adequate power will be obtained to detect the statistical significance. As shown in Fig. 2 below, based on a simulation study with 100 replications, the empirical power for 26 clusters of sizes 6 and 7 are estimated to be 91% and 97%, respectively, which are satisfactory.

### What factors influenced eligibility and what proportion of those approached were eligible?

#### Practice eligibility

All practices (*n* = 239) registered in NI were assessed for eligibility, whereas for logistical reasons practice recruitment in ROI was restricted to the counties of Sligo and Donegal (*n* = 50). Of the 289 GP practices in NI and ROI screened for eligibility, 7 (2.4%) were not eligible due to being involved in another study, resulting in 282 being deemed eligible and invited to participate.

#### Patient eligibility

Patient eligibility screening was undertaken in 2 stages: initial electronic screening using practice healthcare software followed by manual screening conducted by practice staff. The original protocol plan envisaged inviting sequential tranches of 50 patients until 20 patients per practice had been recruited. This had to be amended to only one round of invites per practice due to pressures posed on the practices by the COVID pandemic.

Of 83,364 patients registered with the 15 practices, 79,489 were excluded by electronic screening, largely due to reasons of age or insufficient number of prescribed medications, resulting in 3875 being potentially eligible to participate (Fig. 1). The first tranche of 1115 patients only were manually screened by practice staff; the remaining 2760 were not. Of those manually screened, 677 were deemed eligible and invited to participate. Therefore, of the 80,604 (83,364 − 2,760) screened, 677 (0.84%) were deemed eligible.

### Was recruitment successful? And did eligible participants (practices and patients) consent? 

Similar to Murphy et al. [29] and Abuhaloob et al. [1], the authors experienced difficulties in distinguishing between recruitment (point 3) and consent (point 4) of the ADePT framework. As the practices and patients that consented were recruited. These are presented here simultaneously.

#### Practice consent and recruitment

From the initial 282 general practices invited, 34 (12%) expressed an interest to participate, of which 17 (6%) later declined due to lack of time, lack of support from practice staff, unable to get a peer within the practice to conduct medication reviews, insufficient potential eligible patients and/or staff shortages. A further 2 (.7%) despite extensions, were unable to comit within the study timeframe. The status of 248 (87.9%) was not known as they did not respond. The remaining (15 - 5.3%) consented and were recruited to participate.

Practice recruitment, while recruiting 15 out of a target of 16 practices (94%), took 6 months, therefore longer than originally anticipated. Research staff observed a higher level of interest from practices who received follow-up telephone calls or face-to-face visits from the research team, or interacted with members of the research team at local professional development meetings, suggesting that these strategies would be necessary in any future trial.

#### Patient consent and recruitment

Of the 677 eligible patients invited to participate, 131 patients (19%) returned completed consent forms and baseline questionnaires. The average number of recruited patients per cluster was 8 (range 3–14)**.**

While the mean number of prescribed medications was 12, 9.9% (*n* = 12) were on < 10 medications. This was attributed to the changing nature of medication prescribing. After due consideration, the Trial Management Committee determined that they would continue in the study.

Overall, participant recruitment was particularly challenging with 121 patients out of a proposed 320 (38%) being recruited, taking 5 months longer than initially anticipated. The failure to meet the target of 20 patient patients per cluster was in part due to the context of the COVID pandemic. Additionally, some GPs indicated that patient research documentation was overly long and complex. Ten patients (8%) were excluded as they responded outside the pre-defined one month response timeframe, a criterion that could be modified in a future trial.

Issues and suggested solutions around recruitment are further discussed in Additional file 1: Recruitment.

### Were participants successfully randomised and did randomisation yield equality in groups?

The profiles of the participating practices and patients, allowing for the relatively small numbers appear broadly similar (see Tables 2 and 3). Control practices were more likely to be rural (43% vs 25%) and have more than 7500 patients (71% vs 12%). Control patients were more likely to be male (61% vs 36% in the intervention arm). Seven practices, four in the ROI with 42 patients and three in NI with 17 patients, were randomised to, received and implemented the MyComrade intervention, representing 49% of total patients recruited.

No significant differences were noted with patients between intervention and control groups.

### Were blinding procedures adequate?

Allocation concealment was performed successfully. Given the nature of the intervention and trial resources, it was not possible to blind those collecting data collection or conducting analyses. The cluster randomisation of practices minimised contamination of control practices and patients though the practice teams were aware they were participating in a trial focusing on appropriate medicines management.

### Did participants adhere to the intervention?

Using completion of the intervention medication review checklist as a proxy indicator of adherence to the intervention, trial fidelity was very good. Fifty-two of 59 (88%) intervention medication review checklists were fully completed, following the process outlined in the training programme. This process was described by one GP as:

“*So the two of us sat down and we just went through the charts. Did about two at a time max depending on how the time allowed. And sometimes we only got through one in the allocated time depending on the complexity of the case. And then we made action plans, wrote them in the notes and then either myself or the other GP kind of the lead for that patient and it was our duty then to follow up on the patient and kind of implement the recommendations that we made from the discussions… that’s how we went about it.*” (GP 5).

The remainder were partially completed 8.5% (*n* = 5) or not completed 3.4% (*n* = 2). Thirty-seven (62%) of checklists were uploaded to the practice healthcare system and/or a record of review being conducted was entered into the participant’s medical notes. From those who recorded the time taken (*n* = 38), 31 (81.5%) reported it took between 10 and 30 min to conduct the medication review, of which *n* = 23 (60% of total number who recorded time) took > 20 min to complete. Nine (23.5%) indicated it took > 45 min.

**Fig. 3:**
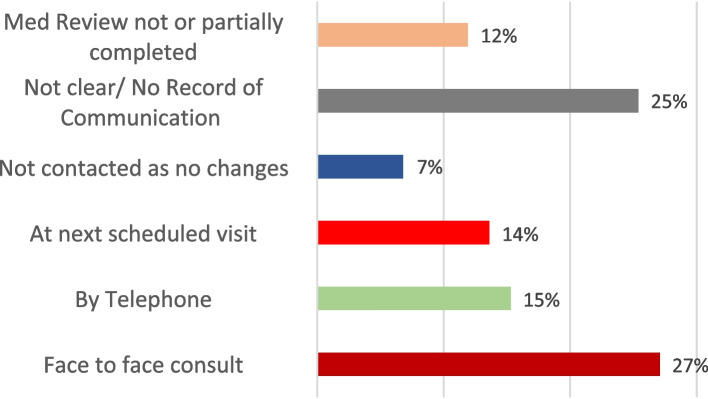
Communication of reviews to patients

The conduct and recommendations from the medication review were found to have been communicated face to face with the participant in 27% (*n* = 16), via a telephone consultation for 15% (*n* = 9) and for 14% (*n* = 8) GPs indicated that the review was to be discussed with the patient at their next scheduled visit. No record of communication was found for 25% (*n* = 15). GPs reported not contacting 7% (*n* = 4) of patients as they deemed this unnecessary due to no medication changes being recommended (see Fig. 3).

### Was the intervention acceptable to participants?

Intervention acceptability was assessed through semi-structured interviews with intervention practitioners (GPs, *n* = 8; PBPs, *n* = 4; and practice administration staff, *n* = 1) (*n* = *13*) and patients (*n* = *12*), on each of the intervention components.

#### Review planning

All HCP participants found the planning of collaborative medication reviews acceptable but making a plan to conduct the reviews with a peer raised challenges, relating to time pressures, juggling of different work priorities and demands compounded by staffing. This was more apparent with the ROI GP:GP dyad as compared to NI GP:PBP dyads. However, practices developed their own solutions to these challenges:

“*We always did it on a day where there’s lots of bodies about so that we wouldn’t get interrupted.*” (GP 4).

Scheduling for two reviewers for the collaborative approach was more challenging for the GP:GP dyad and this meant that the reviews often ended up being conducted on an ad hoc basis when circumstances allowed. One ROI GP (*GP 7*) reported conducting the medication reviews on their scheduled day off as this was the only time when there were two GPs present in the practice.

“*We tried. We’re in the practice three days together so we tried to do them on a Monday or the Tuesday around lunchtime …But you know the way general practice is that didn’t happen all the time.*” (GP 6).

#### Conducting collaborative medication reviews

While acknowledging the additional resource demands, GPs and PBP commended the collaborative approach advocated by the MyComrade intervention, describing that it reduced uncertainty, critically appraised and corroborated treatments plans, increased both practitioner confidence and patient safety.

“*And I think it helps actually instill confidence in the patient you know that there is a second doctor who has looked at their chart so at least there’s two eyes that feel that its safe to stop a medication*.” (GP 5).

“*And it allows me to just reach a more balance decision and a more stimulated thought process than if I was doing it on my own…*” (GP 1).

Similar to GPs and PBPs, patient participants welcomed having two healthcare professionals reviewing their medications. There was a general consensus that peer reviewing was better than sole reviewing as patients believed it would improve the accuracy and appropriateness of treatment regimens:

“*I’m actually very happy about it. Because there’s always a thing there where what one might miss someone else will pick up. And I always say two heads are better than one… So I actually think it’s a very good thing that GPs would just chat or you know go over people’s medication and illnesses and everything else*.” (Patient 1).

#### Use of checklist

Both dyads described the NO TEARS checklist [25] as comprehensive, user friendly and that it provided a standardised approach to medication reviewing. There were some differences noted between how the two dyads used the checklist. The GP:PBP dyad reported using the checklist throughout the study:

“*So that checklist for me was a great skeleton to work from. And we’ve built on that on the day and in the session but it was a great one to keep focusing back to and back to each time that we were going through.*” (GP 4) .

However, the GP:GP dyad indicated that the use of the checklist lessened over time:

“*Yeah we did at the start. After a while you know we didn’t really so much. It was handy to have but I kind of found that we didn’t really need it as much after one or two patients. But it was still good to kind of keep an eye on to make sure that we hadn’t forgotten anything.*” (GP 5).

#### Incentives

ROI GPs expressed that the use of external CPD points (e.g. maintenance of knowledge and skills^i^) was of limited value as an incentive as they had sufficient points from other continuing medical education sources. Suggested alternative incentives included internal CPD points (e.g. practice learning and development), paid study leave or financial compensation for time allocated to the reviews. The responses from the PBPs highlighted the utility of including the medication reviews in annual appraisals:

“*… Yes it would be useful to include as part of our appraisal to say that we’re working on this in the surgery.*” (PBP 2).

Overall, the use of this type of incentive was not viewed as being particularly significant for the HCPs.

The use of financial incentives was addressed throughout the data by many participants. It was quite often linked to opportunity time costs:

“*The payment is fine. I’m happy with the payment. I think it’s probably pitched at the right level. It’s neither an inducement to do it but to not have it would be a detraction. To be honest with you I think if you didn’t get some sort of payment then we probably wouldn’t have engaged*.” (GP 1).

With the GP:GP cohort, the financial incentive aspect was also linked with the feasibility of the intervention becoming part of regular practice:

“*But I think if you were trying to broaden this out I would say it’s very important. And I think okay so if I have the motivation but my partner wasn’t it would be something I could say look you know this is money coming into the practice, we’re not wasting our time here, we can justify the time spent on it.*” (GP 6).

Issues and suggested solutions around incentives are further discussed in Additional file 2: Incentives.

#### Feedback to patients

There was uncertainty amongst some patients about if, or when, a review of their medications had been conducted. A solution to this was evident in the qualitative data, with one GP suggesting that the intervention would benefit from an additional step, having a specific formal consultation discussing the review with the participant:

“*So I think that last piece is tricky and it probably needs a bit more thought about how to actually you know bring the steps to the patient. It’s one thing us knowing it but you have to discuss these things … So that takes another consult in itself to kind of discuss the findings or your recommendations from the discussion… But I just think the last bit was tricky and there’s no point in doing the other bits if you don’t do the last bit*.” (GP 7).

Issues and suggested solutions around patient communication are further discussed in Additional file 3: Communication.

#### Differences between intervention implementation between ROI GP:GP and NI GP:PBP dyads

The difference between the ROI GP:GP and NI GP:PBP dyads contributed to some divergence in perspectives and implementation approaches. GP’s in ROI practices were more concerned about the time required for two GPs to collaboratively review medications outside a research study context. Having PBP input was seen as advantageous by both pharmacists and GPs for two main reasons: because PBPs often reviewed medication lists before meeting the GP thereby saving time within the collaborative review and because they brought pharmacological expertise.

“*So the thoroughness I would say probably of a GP review wouldn’t be to the same standard as our pharmacy colleagues because they’re probably not as pressured and have more allocated time for that sole purpose.*” (GP 4).

“*Well I probably took a bigger role in it than [GP’s name]..So I would have done more of the searching for the indication for the medication, when … why it was started … and took a note of everything and then have a list of things to discuss with them and they would run through them individually with me and take a look themselves and see what they thought. So probably time wise I spent a greater deal of time on it than [GP’s name].*” (PBP 5).

### Was it possible to calculate intervention costs and duration?

Generating the data required for the conduct of a future definitive RCT-based health economic evaluation was feasible (see Appendix 5). In terms of costs, the MyComrade intervention implementation cost per patient was estimated at €490 for ROI and €633 for NI. In terms of total costs at follow-up, the mean estimate for the MyComrade intervention arm was €2573 (SD: 5463) per patient. For the control arm, the equivalent cost estimate was €1362 (SD: 2285). In terms of QALYs gained at follow-up, the mean estimate for the MyComrade intervention arm was 0.45 QALYs (SD: 0.20), compared to 0.39 QALYs (SD: 0.22) for the control arm. A preliminary incremental analysis indicated that the MyComrade intervention was associated with an additional cost of €1211 per patient and additional QALYs of 0.057 per patent relative to the control, which translates into an incremental cost effectiveness ratio of €21,246.A full health economic evaluation would be required to definitively examine the cost effectiveness of the MyComrade intervention.

### Were outcome assessments completed?

#### Practice outcomes: completion of medication reviews

The majority, 88% (*n* = 52), of medication reviews were fully completed and a further 8.5% (*n* = 5) were partially completed; all fully and partially competed reviews were signed by two reviewers and over half (59% *n* = 35) were uploaded into the healthcare system as recommended.

#### Patient outcomes: completion of multimorbidity treatment burden and EQ-5D-3L questionnaires

There were high completion rates of these questionnaires by patient participants at 4 (*n* = 98, 81%) and 8 (*n* = 103, 85%) months (Table 4).

#### Prescribing outcomes: number and changes in prescribed medications, deprescribing and indicators of potentially inappropriate prescribing

Prescribing outcomes were available for all 120 patients (1 patient dropped out due to frailty—see Table 5.

Identification of prescribing information was not straightforward—examples of resolvable but time-consuming issues included incomplete prescriptions, different timeframes for repeat prescriptions and differing search capacities of practice software.

Analysis of prescriptions to identify potentially inappropriate prescriptions [2] revealed 86 instance of PIP. Only four (of twelve) types of PIP’s were identified: No: 2 (asthma and β blockers), No: 3 (ACE inhibitors or diuretics and monitoring of renal function), No 5 (Methotrexate and FBC monitoring) and No: 6 (monitoring of warfarin)(See Appendices 3 and 6). Verifying PIPs were complicated by incomplete histories’ records warranting searching referral letters for supporting background medical history and some laboratory tests being conducted in hospital setting as opposed to primary care, the most problematic being INR for monitoring warfarin effects.

No unintended consequences or effects were identified or reported as a result of the study.

### Were outcomes measured those that were the most appropriate outcomes?

Given that the focus of the MyComrade intervention is on polypharmacy, our chosen outcomes adhere well to those recommended in the COSmm for multimorbidity [47]. They appear appropriate in measuring the responsiveness and impact of the MyComrade intervention and for use in a definitive trial.

### Was retention to the study good?

GP practice retention was 100% with all practices continuing to study end. This was not able given the increased demands on GP practices and their staff during the COVID pandemic.

The majority of patients returned the follow-up questionnaires for the 4 (81% *n* = 98) and 8 (85% *n* = 103) month study time points. One participant, in the intervention group, withdrew prior to any intervention activity due to frailty. Otherwise, reasons for non-return of questionnaires were not identified.

### Were the logistics of running a multicentre trial assessed?

The governance of a trial between two healthcare systems with different ethical and regulatory frameworks posed some challenges, including the development of contracts between the two host universities which took 14 months. This was exacerbated by the withdrawal of the UK from the European Union in January 2020. Initial contact with practices was more straightforward in NI due to the availability of a central register of all practices.

Data collection with multiple general practice software systems was, as expected, complex but feasible with appropriate support from practice staff with software expertise. A full trial therefore may recommend inclusion only of practices with certain healthcare software.

### Did all components of the protocol work well together?

The protocol was delivered as stated with a number of adjustments. These mainly related to the curtailment of patient recruitment due to the global COVID-19 pandemic. Further adjustments for a full trial will be required regarding practice and patient recruitment, practice incentives and appropriate feedback to patients.

#### Progression criteria

We utilised the pre-defined progression criteria [3], originally outlined in the study protocol [23], to further consider how the components of the protocol worked together (Table 7)**.** Two concepts met the ‘Go’ criterion (practice and patient retention), two met the ‘Amend’ criterion (practice recruitment and intervention implementation) and one indicated a ‘Stop – unless changes possible’ (patient recruitment).

**Table 7:**
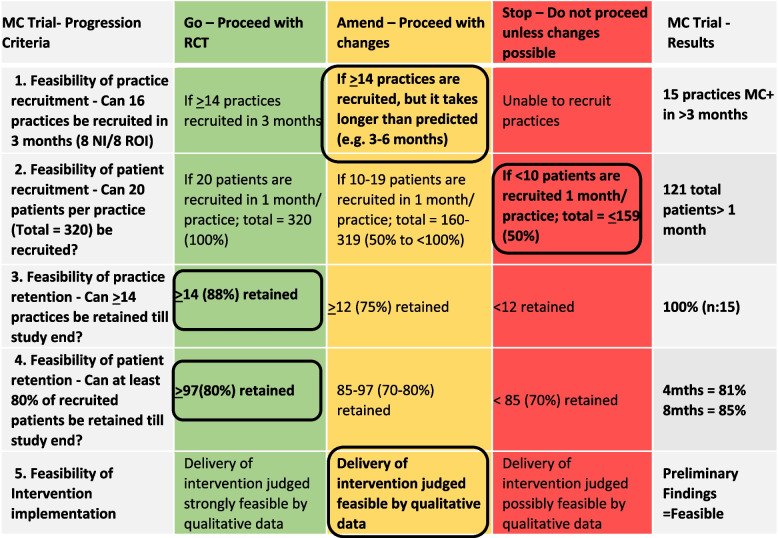
MyComrade progression criteria [23]

Using the ADePT process, the study team developed a number of changes to be incorporated into the design of a definitive trial—see Additional files 1, 2 and 3. These included enhancing recruitment through more interactive recruitment strategies, extending recruitment timeframes, review of incentivisation and consideration to include an additional step, a formalised communication with the patient, to the medication review process.

## Discussion

Feasibility studies [9, 15], supported by the ADePT framework [39], are a critical step in identifying potential problems and solutions when considering progression to a definitive trial. While the MyComrade pilot cluster RCT was, overall, implemented as planned and generated positive feedback, some significant feasibility issues regarding practice and patient recruitment were identified. Other areas identified for consideration before proceeding to a definitive trial included how to both support the opportunity costs of the intervention and ensure optimal communication on the conduct of the medication reviews with patients.

### Recruitment

The onerous nature of recruitment to primary trials [4, 14, 36] is reflected in our findings, with recruitment of practices and in particular patients being challenging and taking longer than expected. The coincidence of this pilot trial with the COVID-19 pandemic exacerbated this problem, with only one round of invites being issued, the potential yield from patients who were not screened or invited is unknown.

Direct interaction with practices is an identified driver for recruitment [36]. We report two different approaches to contacting practices between NI (direct emailing only) and ROI (mix of face-to-face processes)—a combination of these is probably ideal. Complying with GDPR requirements meant that patients were “cold-mailed”, and while contact details of the research team were provided, no enquiries were actually received from any of those invited. In the ROI, an amendment to the Health Research Regulations January 2021 provides an opportunity for designated research staff to conduct the pre-screening of patients for research. Given practice staff find such work challenging, this amendment will be a welcome regulatory change when implemented.

The low patient eligibility rate per practice also contributed to recruitment challenges. The use of the GP practice software systems to identify potential patients is attractive for its simplicity and speed. However, it is entirely reliant on the accurate entry of patient records and prescription data, and software capacity to search the database. As outlined in Additional file 1: Recruitment, possible responses to this problem include lengthening recruitment timeframes, increasing interaction with both practices and patients and utilising only those practice software systems which are compatible with electronic screening.

### Retention

Retention was successful, despite the challenges posed by the COVID pandemic and the vulnerability of the patient cohort.

### Intervention components

HCPs and patients recognised the importance and benefits of medication reviews and the individual components of the intervention appeared to work very well together. The role of incentives to support the opportunity costs of medications reviews is understandably an important one. The importance of acceptable financial imbursement for conducting such reviews was highlighted as critical for their sustainability. Qualitative data suggested less importance for the role of supporting continuing professional development (CPD) requirements; the impact of COVID-19 where such requirements were decreased may have confounded this. Additional file 2: Incentives summarises these issues.

The qualitative data provided real insights into the workings of the GP:GP and GP:PBP dyads. Both worked well; however, the GP:PBP dyad reported advantages in terms of ease of planning, preparatory work being performed by PBP’s, the inherent multi-disciplinary approach and adherence to a checklist approach. Planning a definitive trial will need to closely consider the relative merits of both dyads.

### Communicating the review with patients

Both the Cochrane review (S. M. [44, 46] and NICE guidelines (2017) emphasise the importance of involving patients with multimorbidity and polypharmacy in decisions regarding their medications. The qualitative data highlighted that both patients and healthcare professionals suggested convening a specific formalised consultation between GP or PBP to communicate that a review has occurred and to generate consensus on review findings. This could be incorporated into future modifications of the intervention, but would have additional resource implications. See Additional file 3: Communication.

### Outcomes measures

While the research documentation—information leaflets and questionnaires—for both practices and patient levels was generally well received, some participants did perceive it as unduly lengthy and the completion of questionnaires time consuming. An 85% completion rate of patient questionnaires at 8 months is however quite acceptable, especially in light of population vulnerability and advent of COVID-19.

The authors recognise that this is a pilot trial using a relatively small sample size and that caution needs to be exercised in interpretation of the results. Between control and intervention cohorts from baseline to 8 months, there appeared to be possibly a small change in favour of the intervention group for treatment burden and a tentative trend towards a higher number of medications being discontinued for patients in the intervention group. A definitive trial is clearly required.

#### Strengths and limitations

As recommended by systematic review in this area [45], an inter-professional collaborative intervention was developed according to the MRC Framework (Dieppe, 2008), combined with a strong theoretical framework and incorporated strong public and patient involvement with the potential for economic analyses to link outcomes to costs and benefits. Having qualitative and quantitative evidence added depth and breadth to the evaluation. Rothwell [38] highlighted how frequently generalisability or external validity is overlooked in clinical research. The successful pilot trialling of the MyComrade intervention in two quite different health systems is, from a generalisability perspective, reassuring.

While data were gathered from a smaller number of practices and patients than planned, the numbers recruited are not dissimilar to other pilot cRCTs and while impossible to predict all unforeseen challenges for a bigger trial, some salient ones have been identified here.

Follow-up of eight months is relatively short. COVID impacted on recruitment. When consents were received from patients, they were considered recruited causing difficulty in completion of the ADePT framework [5].

## Conclusions and future directions

We have described the successful piloting of a novel collaborative medication review intervention using a pilot cRCT design in two different primary care systems for persons living with multimorbidity and polypharmacy with a robust process evaluation using both quantitative and qualitative methods.

Despite challenges with practice and patient recruitment, the pilot cRCT demonstrated that a definitive trial of the MyComrade intervention is feasible with amendments. Once recruited, practices and patients did not find participation unduly burdensome, which is creditable considering the vulnerability of the patient population and the context of the Covid-19 pandemic. The trial progression criteria and the ADePT framework facilitated the identification of limitations and potential modifications for a definitive trial.

### Supplementary Information

**Additional file 1.** Recruitment [4, 22, 24, 36, 43, 51, 52].

**Additional file 2.** Incentives [6, 8, 17].

**Additional file 3.** Communication [11, 12, 30, 48].

## Data Availability

NA.

## Appendix 1

**Table 8:** MyComrade outcome variables

Item	Definition	Data source (and reference)
**MyComrade outcome variables—data collection set**		
**PHC practice demographics**		
**PHC location**	Location of GP practice—NI or ROI	Practice Profile Tool
**PHC target Population**	Total number of patients registered with PHC—ROI:(GMS, PP), NI:(NHS)	
**PHC MIS**	GP practice patient management system (e.g. Socrates, HealthOne)	
**PHC setting**	Demographic profile of the PHC clients if urban, rural, mixed based	
**Staffing quota**	PHC Staffing—numbers of full and part time GPs, Trainee GPs, Practice Nurses, Practice Managers and admin staff	
**Staff sessions**	PHC staff sessions for GPs, practice nurses, practice managers and admin	
**Target populations**	Total number of patients registered to practice	
**Repeat prescribing policy document**	Has the practice a repeat prescribing policy? On average how long is spent each day with repeat prescriptions? How are repeat prescriptions requested by patients? (e.g. telephone, e-mail) Who generates repeat prescriptions? (e.g. GP, Admin Staff) Is there a specific time assigned to management of repeat prescriptions? Is the patient’s medical record routinely checked prior to signing the repeat prescription? Do patients in receipt of repeat prescriptions get at least an annual face-to-face medication review?	
**Adverse events policy**	Has the GP practice have a method of reporting/recording adverse event or near misses Yes/No	
**MC + outcomes anonymised participant (patient) level data collected at baseline and 4 and 8 months**		
**Participant (patient) demographics**		
**Date of birth**	Participant date of birth	Patient PHC medical records—Questionnaire Round 1
**Gender**	Sex of participant—male/female/other—describe	
**Marital status**	Participant marital status?	
**Native language**	Language spoken at home—English—Yes/No	
**Education Attainment**	Patients highest level of education	
**Employment Status**	Current job title or title of last paid job	
**Health care Cover**	Participant health cover—if private, public or combined	
**Access to HCP**	Distance patients home is from GP practice Method of travel to GP practice	
**Healthcare utilisation**		
**Patient utilisation of PHC services**	Number of: - GP face to face consultations at practice - GP telephone consultations - GP patient management - GP house calls to participant - GP out of hours visits to participant - Repeat prescriptions without GP face to face consultation - Practice Nurse face to face consultations - Practice Nurse telephone consultations - Practice Nurse management session by GP - Outpatient (OPD) visits - Emergency department presentations (i.e. not admitted) - Hospital admissions—day cases (i.e. discharged same day) - Hospital admissions—inpatient nights ( i.e. overnight stays)	Patient PHC medical records—MyComrade Prescribing Outcomes Tool—Baseline, Round 1 & 2
**Potential inappropriate prescribing**		
**Potential inappropriate prescribing**	Has the patient any of the following PIP and how many of each? - PIP 1: Has the patient a medical history of ever or currently having a peptic ulcer? If yes, are they currently prescribed a NSAID drug? Are they currently prescribed a PPI? - PIP 2: Has the patient a medical history of asthma? If yes, has the patient a medical history of coronary heart disease? If yes are they currently prescribed a Beta Blocker? - PIP 3: For patients aged 75 years or older—has this patient been prescribed an angiotensin converting enzyme inhibitor or a loop diuretic long-term? If yes, has a eGFR and/or ACR being taken in the past 6 months? - PIP 4: Does this patient have a prescription for the combined oral contraceptive pill? If yes, has the patient a medical history of venous or arterial thrombus - PIP 5: Are they currently prescribed a Methotrexate for at least the last 3 months? If yes, has a Full Blood Count being taken in the past 3 months - PIP 6: Are they currently prescribed a Warfarin methotrexate for at least the last 3 months? If yes, has an International Ratio being taken in past 3 months? - PIP 7: Are they currently prescribed Lithium for at least the last 3 months? If yes, Has a lithium level been taken in past 6 months? - PIP 8: Are they currently prescribed Amiodarone for at least 6 month? If prescribed amiodarone for at least 1 month, are they receiving a dose of more than 200 mg per day? If yes what dosage? Has a TFTt been taken in past 6 months?	Patient PHC medical records—Potential Inappropriate Prescribing [2]—MyComrade Prescribing Outcomes Tool—Baseline, Round 1 & 2
**Medication prescribing history**		
**Patient PHC medication prescribing history**	Number of repeat medication prescribed per patient across study timeline	Patient PHC medical records—MyComrade Prescribing Outcomes Tool—Baseline, Round 1 & 2
	Number of repeat medication commenced and discontinued per patient across study timeline	
	Number of repeat medication deprescribed per patient across study timeline	
**EQ-5D-3L—Quality of Life Questionnaire**		
**Self-reported assessment on burden of care**	Self-reported assessment of own quality of life that best describe health state in relation to - Mobility - Performing self-care - Performing usual activities - Having pain & discomfort - Having anxiety and/or depression - Rating health and well being	Self-reported—EQ5D Tool[16]—Baseline, Round 1 & 2 Questionnaires
**Multimorbidity Treatment Burden Questionnaire (MTBQ)**		
**Self-reported assessment on medical treatment burden**	Self-reported assessment on difficulty in: - Taking lots of medications - Remembering how and when to take medications Paying for prescriptions, over the counter medicines or equipment - Collecting prescription medication - Monitoring your medical conditions (e.g. checking your blood pressure or blood sugar) - Arranging appointments with health professionals - Seeing lots of different healthcare professionals - Attending appointments with health professionals (e.g. getting time off work, arranging transport) - Getting health care in the evenings at weekends - Getting help from community services (e.g. Physiotherapists, Public Health Nurse) - Obtaining clear and up to date information about your condition - Making recommended lifestyle changes (e.g. diet, exercise) - Having to rely on help from family and friends	Self-reported—Multimorbidity Treatment Burden Questionnaire (MTBQ; [13]—Baseline, Round 1 & 2 Questionnaires
**Process evaluation data**		
**Key stakeholder perspectives**	Key Stakeholder—GPs and patients’ perspectives on living with multimorbidity, MC + intervention and research processes	Qualitative interview transcripts

## Appendix 2

**Table 9:** The TIDieR (Template for Intervention Description and Replication) checklist

	**Item**	**Where located in MyComrade Trial protocol **[23]
**BRIEF NAME**		
**1**	MultimorbiditY COllaborative Medication Review And DEcision Making (MyComrade)	Page 1
**WHY**		
**2**	We used the results of a systematic review and qualitative interview together with the Capability- Opportunity-Motivation-Behaviour (COM-B) model of behaviour, the Behaviour Change Wheel approach to intervention development and the Behaviour Change Technique (BCT) taxonomy to develop this intervention specifically to facilitate the conduct of active medication review. Based on the findings of a feasibility study, one additional BCT was added to address the need for additional supports to complete medication reviews	Page 5 and http://www.implementationscience.com/content/10/1/132; https://pilotfeasibilitystudies.biomedcentral.com/articles/10.1186/s40814-017-0129-8
**WHAT**		
**3**	Pairs of General Practitioners (GPs) in the Republic of Ireland (ROI), and a GP and Practice-Based Pharmacist (PBP) in Northern Ireland (NI). GPs/GP and PBPs received a brief information session and were provided with instructions on how to implement the intervention. The instructions detailed the six behaviour change techniques included in the intervention. They were also provided with a booklet containing copies of a medication review checklist. This checklist was a modified version of the NO TEARS tool for medication review	GP participant information leaflet https://drive.google.com/file/d/1-3S2nK5HZjADEW04n01NCny-oJxdquvL/view?usp=sharing Instructions for participating GPs/PBPs https://drive.google.com/file/d/12sjxYC9sqsq4NAdvBNZz0GUSnenGKPCT/view?usp=sharing Medication review checklist https://drive.google.com/file/d/1_Y4QczlLgLykiDSCHc2jJpfHVvI3fMYe/view?usp=sharing
**4**	Each pair of GPs or GP and PBP was asked to conduct 20 medication reviews using the MyComrade approach. Patients who were prescribed 10 or more medications were recruited from each practice	Page 9–13
**WHO PROVIDED**		
**5**	Only practicing GPs/PBP implemented the intervention	Page 13
**HOW**		
**6**	GPs or GP and PBP implemented the intervention in pairs	Page 13
**WHERE**		
**7**	The intervention was implemented in the GP practice. The participating GPs, or GP and PBP were asked to come up with an action plan in which they would specify when and where they would conduct the reviews	Page 13
**WHEN & HOW MUCH**		
**8**	Each pair of GPs, or GP and PBP was asked to conduct 20 medication reviews using the MyComrade approach. Patients were recruited from each practice who were prescribed 10 or more medications.They were asked to complete the reviews within a four month interval	Page 9–13
**TAILORING**		
**9**	Participating GPs, or GP and PBP were advised that they could adapt the action plan (when, where, how many patients to review in one sitting etc.) to suit their own practice. This adaption was captured in qualitative interviews	Page 11, 18 and participant information sheets as above
**MODIFICATIONS**		
**10.** ^**ǂ**^	As described above, the original intervention was modified based on findings from a feasibility study. Further modifications were made to account for contextual differences between the two jurisdictions involved in the trial – principally that the reviews were conducted by GP pairs in ROI and GP-PBP pairs in NI. Changes were also made due to the COVID pandemic such as inviting only one tranche of 50 patients per practice	Page 14 and https://pilotfeasibilitystudies.biomedcentral.com/articles/10.1186/s40814-017-0129-8
**HOW WELL**		
**11**	Intervention adherence will be assessed in qualitative interviews	Page 18
**12.** ^**ǂ**^	Observation or recording of implementation of the intervention was not performed	

^**^ Authors—use N/A if an item is not applicable for the intervention being described. Reviewers—use ‘?’ if information about the element is not reported/not sufficiently reported

^†^If the information is not provided in the primary paper, give details of where this information is available. This may include locations such as a published protocol other published papers (provide citation details) or a website (provide the URL)

ǂIf completing the TIDieR checklist for a protocol, these items are not relevant to the protocol and cannot be described until the study is complete

^*^We strongly recommend using this checklist in conjunction with the TIDieR guide (see *BMJ* 2014;348:g1687) which contains an explanation and elaboration for each item

^*^ The focus of TIDieR is on reporting details of the intervention elements (and where relevant, comparison elements) of a study. Other elements and methodological features of studies are covered by other reporting statements and checklists and have not been duplicated as part of the TIDieR checklist. When a randomised trial is being reported, the TIDieR checklist should be used in conjunction with the CONSORT statement (see www.consort-statement.org) as an extension of Item 5 of the CONSORT 2010 Statement. When a clinical trial protocol is being reported, the TIDieR checklist should be used in conjunction with the SPIRIT statement as an extension of Item 11 of the SPIRIT 2013 Statement (see www.spirit-statement.org). For alternate study designs, TIDieR can be used in conjunction with the appropriate checklist for that study design (see www.equator-network.org)

## Appendix 3

**Table 10:** Indicators of potentially inappropriate prescribing (PIP) [2]

	**Outcome**
**Primary**	1. Patients with a history of peptic ulcer who have been prescribed a non-selective non-steroidal anti-inflammatory drug without co-prescription of a proton-pump inhibitor
	2. Patients with asthma who have been prescribed a β blocker
**Secondary**	3. Patients aged 75 years and older who have been prescribed an angiotensin converting enzyme inhibitor or a loop diuretic long-term who have not had a computer-recorded check of their renal function and electrolytes in the previous 15 months
	2a.Patients with asthma (and no history of coronary heart disease) who had been prescribed a β blocker
	4. Proportions of women with a past medical history of venous or arterial thrombosis who had been prescribed the combined oral contraceptive pill
	5. Patients receiving methotrexate for at least 3 months who had not had a full blood count recorded 5a or liver function test 5b in the previous 3 months
	6. Patients receiving warfarin for at least 3 months who had not had a recorded check of their international normalised ratio in the previous 12 weeks
	7. Patients receiving lithium for at least 3 months who had not had a recorded check of their lithium concentrations in the previous 3 months
	8. Patients receiving amiodarone for at least 6 months who had not had a thyroid function test in the previous 6 months
	9. Patients receiving prescriptions of methotrexate without instructions that the drug should be taken every week
	10. Patients receiving prescriptions of amiodarone for at least 1 month who are receiving a dose of more than 200 mg per day Composite secondary outcome measures
**Composite secondary outcome measures**	11. Patients with at least one prescription problem (a combination of outcome measures 1, 2, or 4)
	12. Patients with at least one monitoring problem (a combination of outcome measures 3, 5, 6, 7, and 8) In the trial protocol

## Appendix 4

**Table 11:** CONSORT 2010 checklist of information to include when reporting a pilot or feasibility trial

Section/Topic	Item No	Checklist item		Reported on page No
**Title and abstract**				
	1a	Identification as a pilot or feasibility randomised trial in the title		2
	1b	Structured summary of pilot trial design, methods, results, and conclusions (for specific guidance see CONSORT abstract extension for pilot trials)		2,3
**Introduction**				
Background and objectives	2a	Scientific background and explanation of rationale for future definitive trial, and reasons for randomised pilot trial		4
	2b	Specific objectives or research questions for pilot trial		4
**Methods**				
Trial design	3a	Description of pilot trial design (such as parallel, factorial) including allocation ratio		5
	3b	Important changes to methods after pilot trial commencement (such as eligibility criteria), with reasons		n/a
Participants	4a	Eligibility criteria for participants		5,6
	4b	Settings and locations where the data were collected		5
	4c	How participants were identified and consented		5,6
Interventions	5	The interventions for each group with sufficient details to allow replication, including how and when they were actually administered		6,7
Outcomes	6a	Completely defined prespecified assessments or measurements to address each pilot trial objective specified in 2b, including how and when they were assessed		8
	6b	Any changes to pilot trial assessments or measurements after the pilot trial commenced, with reasons		n/a
	6c	If applicable, prespecified criteria used to judge whether, or how, to proceed with future definitive trial		6,7
Sample size	7a	Rationale for numbers in the pilot trial		n/a
	7b	When applicable, explanation of any interim analyses and stopping guidelines		n/a
Randomisation:				
Sequence generation	8a		Method used to generate the random allocation sequence	10
	8b		Type of randomisation(s); details of any restriction (such as blocking and block size)	10
Allocation concealment mechanism	9		Mechanism used to implement the random allocation sequence (such as sequentially numbered containers), describing any steps taken to conceal the sequence until interventions were assigned	10
Implementation	10		Who generated the random allocation sequence, who enrolled participants, and who assigned participants to interventions	10
Blinding	11a		If done, who was blinded after assignment to interventions (for example, participants, care providers, those assessing outcomes) and how	n/a (19)
	11b		If relevant, description of the similarity of interventions	n/a
Statistical methods	12	Methods used to address each pilot trial objective whether qualitative or quantitative		13,17,18
**Results**				
Participant flow (a diagram is strongly recommended)	13a	For each group, the numbers of participants who were approached and/or assessed for eligibility, randomly assigned, received intended treatment, and were assessed for each objective		13,17,18
	13b	For each group, losses and exclusions after randomisation, together with reasons		13
Recruitment	14a	Dates defining the periods of recruitment and follow-up		5
	14b	Why the pilot trial ended or was stopped		n/a
Baseline data	15	A table showing baseline demographic and clinical characteristics for each group		11,12
Numbers analysed	16	For each objective, number of participants (denominator) included in each analysis. If relevant, these numbers should be by randomised group		14
Outcomes and estimation	17	For each objective, results including expressions of uncertainty (such as 95% confidence interval) for any estimates. If relevant, these results should be by randomised group		14,24–27
Ancillary analyses	18	Results of any other analyses performed that could be used to inform the future definitive trial		21–24
Harms	19	All important harms or unintended effects in each group (for specific guidance see CONSORT for harms)		25
	19a	If relevant, other important unintended consequences		n/a
**Discussion**				
Limitations	20	Pilot trial limitations, addressing sources of potential bias and remaining uncertainty about feasibility		29,30
Generalisability	21	Generalisability (applicability) of pilot trial methods and findings to future definitive trial and other studies		28,30

## Appendix 5

**Table 12:** MyComrade programme cost estimates

	**Republic of Ireland**	**Northern Ireland**	
	***N*** ** = 42**	***N*** ** = 17**	
**Category**	**Euro**	**Stg**	**Euro**
**Site recruitment travel and time**	€2614	£472	€553
**Site initiation travel and time**	€6071	£2555	€2991
**Intervention travel and time**	€3106	£1741	€2038
**Initiation and set up allocation for GP practice**	€2000	£1720	€2013
**Intervention training and conduct of Medication Reviews**	€2500	£1075	€1258
**GP Medication review**	€4196	£1530	€1791
**Folders and packs (all stages)**	€114	£99	€116
**Total**	**€20,600**	**£9,193**	**€10,761**
**Average cost per patient**	**€490**	**£541**	**€633**
**Sterling prices were converted as appropriate using Purchasing Power Parity (PPP) index as per HIQA guidelines**			

**Table 13:** EQ-5D-5L utility score estimates at baseline and follow-up

	***Intervention*** ***N*** ** = ** ***59***	***Control*** ***N*** ** = ** ***62***	***Intervention*** ***N*** ** = ** ***59***	***Control*** ***N*** ** = ** ***62***	***Intervention*** ***N*** ** = ** ***59***	***Control*** ***N*** ** = ** ***62***
*Health outcomes*	Baseline, *mean (SD)*		4 months, *mean (SD)*		8 months, *mean (SD)*	
*EQ-5D-5L Score*	0.67 (0.29)	0.52 (0.40)	0.66 (0.30)	0.58 (0.36)	0.70 (0.29)	0.56 (0.35)

**Table 14:** Health utilisation resource use costing estimates at baseline and follow-up

	**Intervention**	**Control**	**Intervention**	**Control**	**Intervention**	**Control**
	Baseline, *mean (SD)*		4 months, *mean (SD)*		8 months, *mean (SD)*	
**Resource items**						
**GP visits:**	2.04 (1.89)	1.65 (2.23)	1.70 (1.97)	1.25 (1.64)	1.35 (1.51)	1.33 (1.75)
**PN visits**	1.35 (1.49)	1.17 (1.77)	1.47 (1.69)	1.10 (2.28)	1.04 (1.07)	1.79 (2.21)
**A&E visits**	0.21 (0.65)	0.21 (0.65)	0.16 (0.53)	0.22 (0.61)	0.09 (0.29)	0.19 (0.47)
**Outpatient visits**	1.37 (1.32)	1.08 (1.35)	1.30 (1.35)	1.08 (1.53)	0.77 (0.95)	0.97 (1.49)
**Inpatient days**	0.04 (0.19)	0.05 (0.21)	0.09 (0.29)	0.08 (0.33)	0.07 (0.26)	0.13 (0.34)
**Inpatient nights**	0.26 (1.47)	1.10 (6.64)	1.14 (5.45)	0.37 (1.20)	0.23 (0.91)	0.44 (1.86)
**Total healthcare cost converted to Euro €**	639.96 (1299.84)	1363.33 (6354.69)	1535.00 (5307.97)	636.35 (1086.76)	509.96 (992.22)	725.32 (1556.01)

*GP* General practitioner, *PN* Practice nurse, *A&E* Accident and emergency

Completeness of data:

Intervention: *Baseline –* 2% missing data for GP visits, Practice nurse visits, A&E visits, outpatient visits, inpatient days, inpatient nights and EQ-5D-5L

Control: *Baseline* –0% missing data for GP visits, Practice nurse visits, A&E visits, outpatient visits, inpatient days, inpatient nights and 5% missing on EQ-5D-5L

Intervention: *Follow-up (Time 2) –* 2% missing data for GP visits, Practice nurse visits, A&E visits, outpatient visits, inpatient days, inpatient nights and 26% for EQ-5D-5L

Control: *Follow-up (Time 2)*–0% missing data for GP visits, Practice nurse visits, A&E visits, outpatient visits, inpatient days, inpatient nights and 22% missing on EQ-5D-5L

Intervention: *Follow-up (Time 3) –* 2% missing data for GP visits, Practice nurse visits, A&E visits, outpatient visits, inpatient days, inpatient nights and 22% for EQ-5D-5L

Control: *Follow-up (Time 3)* –0% missing data for GP visits, Practice nurse visits, A&E visits, outpatient visits, inpatient days, inpatient nights and 11% missing on EQ-5D-5L

**Table 15:** Economic cost analysis outcomes—categories of unit cost estimates in 2020 prices

Healthcare Resources	**Activity**	**Republic of Ireland**		**Northern Ireland**		**STG £ converted to € euro using PPP**
		**Unit Cost €**	**Source**	**Unit Cost £**	**Source**	
GP visits:	Per visit	€52	[44, 46]	£33	PSSRU	€39
PN visits	Per visit	€41	Study Records	£10	PSSRU	€11
A&E visits	Per visit	€289	HPO	£222	PSSRU	€260
Outpatient visits	Per visit	€140	HPO	£135	PSSRU	€158
Inpatient days	Per day	€747	HPO	£752	PSSRU	€880
Inpatient nights	Per night	€962	HPO	£602	PSSRU	€705

*GP* General practitioner, *PN* Practice nurse, *A&E* Accident and emergency, *HPO* Healthcare Pricing Office Admitted Price List. Where necessary unit costs were inflated using the health component of the consumer price index from the Central Statistics Office. (PSSRU) Personal Social Services Research Unit in UK. Sterling prices were converted as appropriate using Purchasing Power Parity (PPP) index as per HIQA guidelines

**Table 16:** Incremental cost-effectiveness results—univariate analysis

***Variable/analysis***	***Incremental analysis*** * (intervention minus control)*	
	**Intervention**	**Control**
***Cost analysis***		
* Mean total cost (SD)*	2572.60 (5463.10)	1361.67 (2285.11)
* Difference in mean total cost € (95% CIs), [p-value]*	1211, (− 278, 2699), [0.110]	
***Health outcome analysis***		
* Mean QALYS (SD)*	0.45 (0.20)	0.39 (0.22)
* Difference in mean QALYs, (95% CIs), [p-value]*	0.057, (− 0.036, 0.150), [0.224]	

*CI* Confidence intervals

**Table 17:** Proportion of responses by level of severity for EQ-5D-5L dimensions at baseline and at 8 month follow-up by treatment

		Intervention			Control		
**Dimensions**	**Levels**	**Baseline** ***N*** ** = 59** **%**	**Follow-up** **8 months** ***N*** ** = 46** **%**	**Difference baseline to follow-up time**	**Baseline** ***N*** ** = 62** **%**	**Follow-up** **8 months** ***N*** ** = 57** **%**	**Difference baseline to follow-up time**
Mobility							
	**None**	38.60	36.96	− 1.64	19.35	26.32	6.97
	**Slight**	26.32	23.91	− 2.41	29.03	22.81	− 6.22
	**Moderate**	24.56	28.26	3.70	35.48	35.09	− 0.39
	**Severe**	10.53	10.87	0.34	11.29	14.04	2.75
	**Unable**	0.00	0.00	0.00	4.84	1.75	− 3.09
Self-care							
	**None**	75.44	62.22	− 13.22	61.29	56.14	− 5.15
	**Slight**	8.77	26.67	17.90	12.90	21.05	8.15
	**Moderate**	15.79	8.89	− 6.90	16.13	17.54	1.41
	**Severe**	0.00	2.22	2.22	1.61	1.75	0.14
	**Unable**	0.00	0.00	0.00	8.06	3.51	− 4.55
Usual activities							
	**None**	38.60	32.61	− 5.99	20.97	26.32	5.35
	**Slight**	24.56	32.61	8.05	33.87	31.58	− 2.29
	**Moderate**	24.56	26.09	1.53	24.19	19.30	− 4.89
	**Severe**	10.53	6.52	− 4.01	11.29	12.28	0.99
	**Unable**	1.75	2.17	0.42	9.68	10.53	0.85
Pain/discomfort							
	**None**	19.30	31.11	11.81	9.84	17.54	7.70
	**Slight**	26.32	13.33	− 12.99	31.15	22.81	− 8.34
	**Moderate**	33.33	44.44	11.11	32.79	35.09	2.30
	**Severe**	17.54	6.67	− 10.87	21.31	21.05	− 0.26
	**Extreme**	3.51	4.44	0.93	4.92	3.51	− 1.41
Anxiety/depression							
	**None**	50.88	52.17	1.29	45.00	37.50	− 7.50
	**Slight**	24.56	26.09	1.53	26.67	30.36	3.69
	**Moderate**	24.56	19.57	− 4.99	20.00	30.36	10.36
	**Severe**	0.00	2.17	2.17	8.33	1.79	− 6.54
	**Extreme**	0.00	0.00	0.00	0.00	0.00	0.00

## Appendix 6

**Table 18:** Potentially inappropriate prescribing (PIPs)

	**Control**	**Intervention**	**Total**	
No of PIPs	**(** ***N*** ** = 46)**	**(** ***N*** ** = 40)**	**(** ***N*** ** = 86)**	
Baseline—Number of PIP 2	**5**	**8**	13	15%
4 months—Number of PIP 2	**5**	**7**	12	14%
8 months—Number of PIP 2	**5**	**7**	12	14%
Baseline—Number of PIP 3	**12**	**7**	19	22%
4 months—Number of PIP 3	**9**	**4**	13	15%
8 months—Number of PIP 3	**7**	**7**	14	16%
Baseline—Number of PIP 5	**0**	**0**	0	0%
4 months—Number of PIP 5	**0**	**0**	0	0%
8 months—Number of PIP 5	**1**	**0**	1	1%
Baseline—Number of PIP 6	**1**	**0**	1	1%
4 months—Number of PIP 6	**0**	**0**	0	0%
8 months—Number of PIP 6	**1**	**0**	1	1%
