# Supplementary material for: The multimorbidity collaborative medication review and decision making (MyComrade) study: a pilot cluster randomised trial in two healthcare systems
Source: Pilot Feasibility Stud. 2022 Oct 4;8:225. doi: 10.1186/s40814-022-01107-y (PMC9531225; doi:10.1186/s40814-022-01107-y)
Supplement: Supplementary file 1 — Additional file 1. Recruitment [4, 22, 24, 36, 43, 51, 52]. [file 40814_2022_1107_MOESM1_ESM.docx]

**Additional File 1 – Recruitment**

| STEP 1: PROBLEM TYPE | | | |  |
| --- | --- | --- | --- | --- |
| **TYPE A**: There was a problem with cluster and participant recruitment. Issue likely to be a problem ONLY FOR THE TRIAL (i.e. unlikely to be a problem in the real world) | | | |  |
| **EVIDENCE**   1. 15 out of a target of 16 practices recruited (94%), but took longer than anticipated. 2. 121 out of a target of 320 patients recruited, prior to cluster randomisation, taking longer than anticipated 1 month (38%). | | | |  |
| STEP 2: SOLUTIONS | | | |  |
| **CHANGE ASPECTS OF:**  **a) INTERVENTION**  n/a  **b) TRIAL DESIGN**   1. Review recruitment timeframes allocated to recruitment of clusters and participants – include *additional mail outs, additional recruitment rounds* 2. *Increase interactivity* as part of recruitment process    1. Cluster Level – Follow-up email invites with phone calls, present study face to face or virtually with GPs at practice, at trainings or meetings    2. Patient Level – promote participant engagement with research team and/or GP practice as part of recruitment, increase visibility of study through posters in GP practices and pharmacists 3. *PPI and relevant trial management committee members involvement* with the review of documentation and process with the aim to reduce and simplify without lessening quality, the trial documentation and processes 4. Access expert input into development of *bespoke search strategies* that can be used with different health information systems to optimise identification of eligible participants. Only recruit practices with information systems which support electronic patient identification.   **c) CONTEXT**  n/a | | | |  |
| STEP 3: ASSESSMENT OF SOLUTIONS (TRIAL DESIGN) | | |  |  |
| Could solution b1 be **effective** in a trial setting? **YES** | Could solution b2 be **effective** in a trial setting? **YES** | Could solution b3 be **effective** in a trial setting? **YES** | Could solution b4 be **effective** in a trial setting? **UNSURE** |  |
| **EVIDENCE:** *Additional mail outs and recruitment rounds:* One mail out of a sampling frame of 677 patients resulted in 121 of proposed 320 patients. An additional 2,760 potential participants were not invited. Given time, further rounds of invites may have resulted in the proposed patient numbers. | **EVIDENCE**: *Increased interactivity:*  Of 44 practices approached in RoI, using a more interactive but more resource demanding (cost and GP time) approach, 8 consented within reasonable timeframes, a further 12 engaged but declined later and 24 did not respond. In NI, of 238 practices emailed (less costly and less time consuming), 7 consented, a further 7 engaged of which 5 later declined and 2 deemed ineligible, 224 did not respond).  Evidence shows that while sufficient time is very important, it is not the only factor that influences GP participating in research. Interaction which includes involving GP with the research process, evoking an interest and demonstrating positive impact for patient care motivate GPs interest in research (Bower, 2009; Hummers-Pradier et al., 2008; Richell, 2020).  Interactivity with participants is also a key recruitment factor with patients (Richell, 2020; Sluggett et al., 2021; van den Brink et al., 2020), where patients knowledge and understanding of the study, relevancy to their disease burden and communication either through GP or research team can all have a positive influencing in engagement and participation. | **EVIDENCE**: *Trial documentation and processes – clear and comprehensive*  Clear, simple and comprehensive documentation and processes are critical in research design and can most appropriately be influenced by those who have insight and experience from both provider and user perspective (Bower, 2009; van den Brink et al., 2020; Ward & Bower, 2010). Evidence shows that PPI (INVOLVE, 2016) and trial management committee members are instrumental in informing research design. While both these concepts were integrated into the research design and implementation, this aspect could be further supported and enhanced in roll out of a definitive trail | **EVIDENCE**: *Bespoke MIS search strategies.*  Relying on healthcare software search strategies is complex and requires specific focus and more in-depth exploration. |  |
| Step 4: EVALUATION OF SOLUTIONS | | | | |
| BOX 1: OPTIONS THAT SHOULD WORK IN TRIAL CONTEXT | | | | |
| **Stage 1: Options (ranked by likely feasibility & effectiveness**   1. Increase time frame to allow multiple invites to cluster and participant recruitment 2. Increase interactivity as part of recruitment process 3. Review, with aim to reduce and simplify, the trial documentation 4. Consider seeking expert input into development of bespoke search strategies that can be used with different health information systems to optimise identification of eligible participants | | | | |
| **Stage 2: Potential to combine solutions**  Yes possible to combine 1, 2 and potentially 3 | | | | |
| **Stage 3: Most cost effective solutions**  The listed solutions are easily incorporated into the trial design, and it should be possible to fund them appropriately to ensure any definitive trial is successful. | | | | |
