# Supplementary material for: The multimorbidity collaborative medication review and decision making (MyComrade) study: a pilot cluster randomised trial in two healthcare systems
Source: Pilot Feasibility Stud. 2022 Oct 4;8:225. doi: 10.1186/s40814-022-01107-y (PMC9531225; doi:10.1186/s40814-022-01107-y)
Supplement: Supplementary file 2 — Additional file 2. Incentives [6, 8, 17]. [file 40814_2022_1107_MOESM2_ESM.docx]

**Additional File 2 Incentives**

| STEP 1: PROBLEM TYPE | |
| --- | --- |
| **TYPE C**: Health professionals, particularly GPs, stated that the time required to conduct collaborative medication reviews could be problematic influencing intervention generalisability and feasibility outside of a trial. | |
| **EVIDENCE**:  Throughout the qualitative data there are references to or was stated by the health professionals that the time required to conduct the medication reviews collaboratively may impact on the intervention feasibility and generalisability. | |
| STEP 2: SOLUTIONS | |
| **CHANGE ASPECTS OF:**  **a) INTERVENTION**  1. Enhanced incentivisation and externally supported practice based pharmacists, as in NI  2. Emphasise benefits of collaborative reviews during intervention training / briefing  **b) TRIAL DESIGN**  1. Enhanced incentives and externally supported practice based pharmacists, as in NI  2. Emphasise benefits of collaborative reviews during intervention training / briefing  **c) CONTEXT**  Not applicable | |
| STEP 3: ASSESMENT OF SOLUTIONS | |
| Could solution a1be **effective** in a trial setting? **YES** | Could solution a2* be **effective** in a trial setting? **YES** |
| **EVIDENCE**: Incentives, such as Pay For Performance, for additional workload are a well-recognised and often utilised methodology within work and organisational psychology models (Cadsby et al., 2007; Fang & Gerhart, 2012). | **EVIDENCE**: By emphasising the, non- monetary, benefits to both HCPs and patients the intrinsic motivation to conduct collaborative medication reviews will increase the use of the intervention. |
|  |  |
| Could solution a1 be **feasible** in a real world setting? **YES** | Could solution a2 be **feasible** in a real world setting? **YES** |
| **EVIDENCE**: Adequately resourcing GPs for conducting collaborative medication reviews is feasible. The experience of practice-based pharmacists in NI is positive. This along with additional and more salient professional development incentives, as highlighted in the qualitative data, will result in the generalisability of the intervention increasing. | **EVIDENCE**: Increasing intrinsic motivation has been shown(Cerasoli et al., 2014) to result in increased creativity and productivity in workplaces. |
| Step 4: Evaluation of Solutions | |
| BOX 1: OPTIONS THAT SHOULD WORK IN TRIAL AND REAL WORLD SETTING CONTEXT | |
| **Stage 1: Options (ranked by likely feasibility & effectiveness**  1: Enhanced incentives  2: Emphasise professional benefits to both HCPs and patients of conducting collaborative medication reviews | |
| **Stage 2: Potential to combine solutions**  1 & 2 could be easily combined | |
| **Stage 3: Most cost effective solutions**  1 & 2 combined | |
