# Supplementary material for: The multimorbidity collaborative medication review and decision making (MyComrade) study: a pilot cluster randomised trial in two healthcare systems
Source: Pilot Feasibility Stud. 2022 Oct 4;8:225. doi: 10.1186/s40814-022-01107-y (PMC9531225; doi:10.1186/s40814-022-01107-y)
Supplement: Supplementary file 3 — Additional file 3. Communication [11, 12, 30, 48]. [file 40814_2022_1107_MOESM3_ESM.docx]

**Additional File 3 Communication**

| STEP 1: PROBLEM TYPE | | | |
| --- | --- | --- | --- |
| **TYPE B**: There was a problem with participant recall of medication review and poor communication by GPs with patients of the medication review.  Issue likely to be a problem FOR THE TRIAL AND THE REAL WORLD | | | |
| Majority of the participants with the intervention group, that were interviewed, had difficulty or did not recall having a medication review as part of MyComrade Trial  Over 32% of medication reviews were not communicated to participants. Reasons for not contacting patients included no changes made to prescription so felt not necessary (6.8% of total reviews), scheduled for next GP visit but had not taken place (13.6% of total reviews).  Participants wanted and recommended post medication review communication as integral part of their medication reviews.  Some GPs suggested having an additional step – a specific formal (paid) consultation with patient as part of medication review, to be added to MyComrade Intervention. | | | |
| STEP 2: SOLUTIONS | | | |
| **CHANGE ASPECTS OF:**  **a) INTERVENTION**   - Include communication strategy as part of medication review process - Include specific formal consultation with patient as part of medication review regardless if medication changes were recommended or not. - GP/PBP to receive reimbursement for this additional consultation as per routine GP/Patient consultation rate   **b) TRIAL DESIGN**   - Include communication strategy as part of medication review process - Include specific formal consultation with patient as part of medication review regardless if medication changes were recommended or not. - GP/PBP to receive reimbursement for this additional consultation as per routine GP/Patient consultation rate     **c) CONTEXT**  n/a | | | |
| STEP 3: ASSESSMENT OF SOLUTIONS (TRIAL DESIGN) | | |  |
| Could solution b1 be **effective** in a trial setting? **YES** | Could solution b2 be **effective** in a trial setting? **YES** | Could solution b1 be **effective** in a real life setting? **UNSURE** | Could solution b2 be **effective** in a real life setting? **UNSURE** |
| **EVIDENCE**:  Evidence from interviews - problems with communication of outcomes of review with  Literature indicated the importance of communicating and collaborating with patients as integral part of med review  (De Bock et al., 2018; Duerden et al., 2013; Muth et al., 2019) | **EVIDENCE**:  Reimbursement of consultations with evidence in literature payment to GP & Pharmacist for conducting medication review followed by paid consultation with patients (Sorensen et al., 2004) | **EVIDENCE**:  Evidence from interviews - problems with communication of outcomes of review with  Literature indicated the importance of communicating and collaborating with patients as integral part of med review  (De Bock et al., 2018; Duerden et al., 2013; Muth et al., 2019) | **EVIDENCE**  Reimbursement of consultations with evidence in literature payment to GP & Pharmacist for conducting medication review followed by paid consultation with patients (Sorensen et al., 2004) |
| Step 4: Evaluation of Solutions | | | |
| BOX 1: OPTIONS THAT SHOULD WORK IN TRIAL AND REAL WORLD CONTEXT | | | |
| **Stage 1: Options (ranked by likely feasibility & effectiveness**   - Include communication strategy - Include additional consultation to communicate medication review - Reimburse GP/PBP for medication review consultation | | | |
| **Stage 2: Potential to combine solutions**  Yes 1, 2 & 3 | | | |
| **Stage 3: Most cost effective solutions**  The listed solutions are easily incorporated into the trial design, and it should be possible to fund them appropriately to ensure any definitive trial is successful. | | | |
